# The cost of implant waste in trauma orthopaedic surgery and sustainability considerations: an observational study

**DOI:** 10.1007/s00264-025-06532-1

**Published:** 2025-04-21

**Authors:** Rasi Mizori, Muhayman Sadiq, Yasser Al Omran, Charmilie Chandrakumar, Thomas Lewis, Omar Musbahi, Karthik Karuppaiah

**Affiliations:** 1 King’s College London, London, UK; 2 Royal Free London NHS Foundation Trust, London, UK; 3 King’s College Hospital NHS Foundation Trust, London, UK; 4 Orthopaedics and Arthritis Specialist Centre, Sydney, Australia; 5 Imperial College London, London, UK

**Keywords:** Trauma and orthopaedics, Implant wastage, Sustainability, Healthcare costs, Environmental impact, Surgical inefficiency, Preoperative planning, Medical devices, Locking screws, Economic burden

## Abstract

**Purpose:**

Implant wastage in trauma and orthopaedic (T&O) surgery remains an under-reported yet significant issue, contributing to rising healthcare costs and environmental concerns. With increasing surgical demand driven by an ageing population and the growing prevalence of conditions like osteoporosis, this study aimed to quantify implant wastage in T&O procedures at a Level 1 Major Trauma Centre in London, assessing both its frequency and financial impact.

**Methods:**

A retrospective cohort study was conducted on all weekday T&O procedures performed between 1st December 2023 and 31st January 2024. Two of the authors identified wasted implants using intraoperative implant logbooks, and cross-referencing implant stickers with post-operative radiographs. Data pertaining to patient demographics, procedure types, surgical sites, and implant usage were collected. Cost analysis was performed using procurement data to determine the financial impact of implant wastage.

**Results:**

Among 184 procedures analysed, 131 (71.2%) used implants, with wastage observed in 108 (82.4%) cases. A total of 141 implants were wasted, with screws accounting for 92.9% (*n* = 131) of wasted implants. Locking screws were the most frequently discarded (*n* = 65; 46.1%). Across ORIF and intramedullary nailing procedures, an overall screw wastage rate of 20% (17–31%) was observed with 2.4 screws wasted per trauma procedure. The financial cost of implant wastage over the 44-day study period amounted to approximately £335 per day and £136 per case.

**Conclusion:**

This study highlights the substantial economic burden associated with implant wastage in T&O surgery, with screws, particularly locking screws, being the primary contributors. Targeted interventions, including improved preoperative planning, precision-based implant selection, and enhanced intraoperative decision-making, are essential to reducing waste and improving cost-efficiency and sustainability in surgical practices. Further research should explore the broader economic and environmental impact of implant wastage, incorporating factors such as operative time and carbon footprint to develop comprehensive waste-reduction strategies.

**Level of evidence:**

IV.

## Introduction

Trauma and orthopaedic (T&O) surgeries account for a significant and growing portion of global healthcare, driven by an ageing population and increasing prevalence of conditions such as osteoarthritis [1]. By 2050, hip fractures alone are projected to reach 4.5 million cases annually worldwide [2]. In the United Kingdom, the National Joint Registry (NJR) reports that over 200,000 joint replacement surgeries are performed annually [3], while in the United States, knee replacements are expected to rise to 3.5 million procedures annually by 2030 [4]. Beyond joint arthroplasty, trauma procedures also impose a large burden on healthcare systems globally. In 2017, the number of orthopaedic surgery procedures performed worldwide totalled approximately 22.3 million, with projections indicating a growth of 4.9% annually [5]. Across both elective and trauma orthopaedic procedures, implants such as plates, screws, and nails are indispensable for restoring function and mobility. However, this reliance on implants brings significant economic and environmental challenges, particularly with the disposal of unused or wasted implants contributing to healthcare costs and carbon emissions [6].

The global orthopaedic implants market, valued at USD 45.19 billion in 2023, is anticipated to grow to USD 71.74 billion by 2032 [7], reflecting the substantial reliance on these devices. In the US, spending on medical devices totalled US $173 billion in 2016, accounting for 5.2% of all national health expenditures [8]. Similar to other industries, T&O is subject to waste and inefficiency. Studies in the UK have identified significant levels of implant wastage within T&O procedures, with a particular study highlighting a 15% increase in operative costs due to screw wastage [9]. Payne et al. reported that 12% of procedures in their department over 12 months involved implant wastage, while Jayakumar et al. found that implant wastage occurred in 25.1% of trauma procedures over a one-year period [8, 10]. Contributing factors include defective components, intraoperative errors, and improper handling, all of which exacerbate financial and environmental strain [11].

Implants, as a major contributor to surgical costs, represent a key area for cost-saving and environmental interventions [6]. Addressing implant waste not only offers a pathway to reduce unnecessary expenditure for hospital systems but also aligns with broader financial recovery strategies. Furthermore, reducing implant wastage could significantly improve environmental practices.

Our study aimed to quantify the implant usage in T&O surgeries at a Level 1 Major Trauma Centre hospital. The primary objective was to calculate the percentage of unused implants out of the total opened. The secondary aim was to estimate the financial cost of implant wastage and identify procedures associated with the highest levels of waste. Finally, we sought to evaluate the broader implications of implant waste on long-term sustainability in T&O care.

## Materials and methods

### Study setting

This study was conducted at a tertiary major trauma centre hospital in London, United Kingdom. The centre has two operating theatres and six trauma and orthopaedic wards, providing care for polytrauma patients.

### Study design

This was a single-centre, retrospective cohort study analysing all consecutive trauma and orthopaedic procedures performed between December 1, 2023, and January 31, 2024. The implant logbooks within each theatre were utilised, which list the respective procedures in the theatre and include product labels for the implants opened in a particular operation. Two authors identified wasteful implants independently through a systematic review of the implant logbooks. Secondary confirmation of implant wastage was confirmed in cases with post-operative imaging to confirm the correct number of implants.

### Data collection and analysis

Data collected from the theatre logbooks included patient demographics, procedure type, site of the procedure and the number and type of implants opened intraoperatively. Product codes for all implants were recorded. Implant wastage was assessed by cross-referencing implant stickers with post-operative radiographs. Instances of implant wastage were determined by comparing the materials implanted with those opened intraoperatively. A procurement hospital spreadsheet detailing manufacturers and costs of various implant sizes was used to calculate the cost of implant wastage. The mean cost per implant type was calculated by averaging the prices from different manufacturers. This average cost was multiplied by the number of wasted implants to determine total and specific implant wastage costs.

### Ethics

The NHS Health Research Authority’s decision tool indicated that this work did not require formal ethical approval as clinical research. Local service evaluation approval was obtained (number: 19062024/12).

## Results

### Implant wastage

**Fig. 1:**
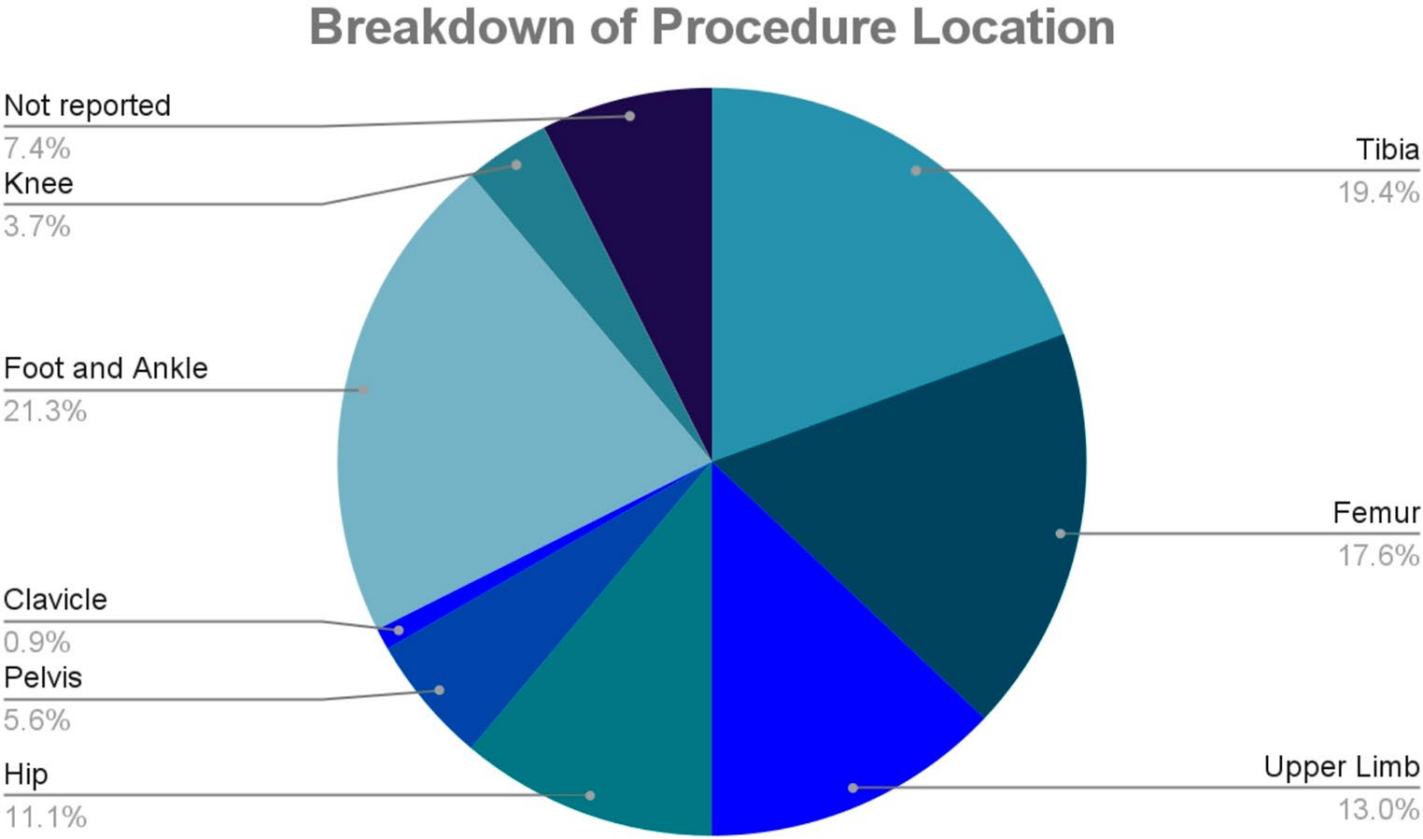
Breakdown of procedure locations in trauma orthopaedic surgery

Between December 1, 2023, and January 31, 2024, a total of 184 trauma procedures were performed. The majority involved the foot and ankle (*n* = 23, 21.3%), the tibia (*n* = 21, 19.4%) and the femur (*n* = 19, 17.6%) (Fig. 1).

**Fig. 2:**
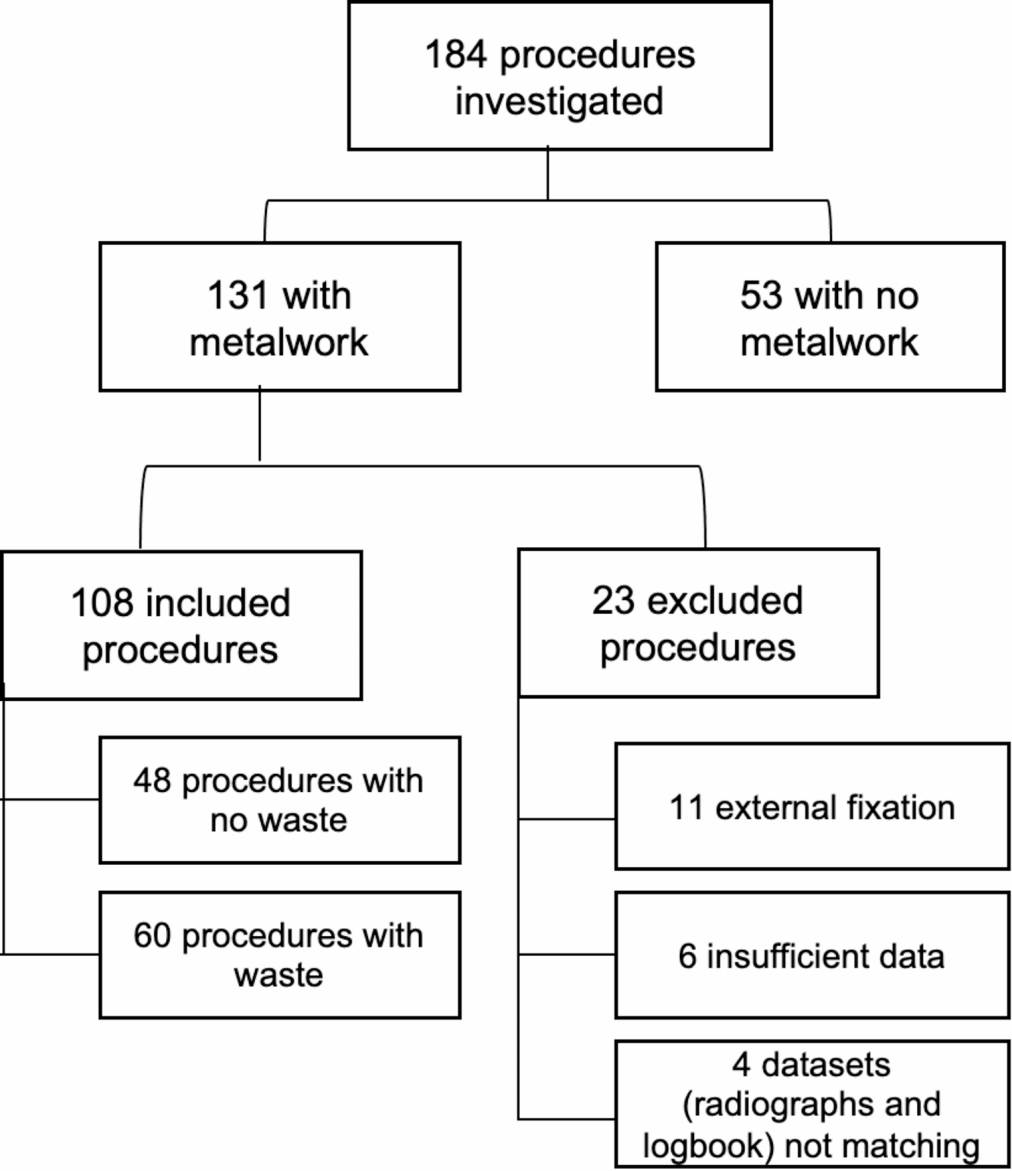
Flowchart illustrating the inclusion for the study

Of these procedures, 131 (71.2%) involved the use of metal implants, with wastage observed in 108 procedures (82.4%) (Fig. 2).

**Fig. 3:**
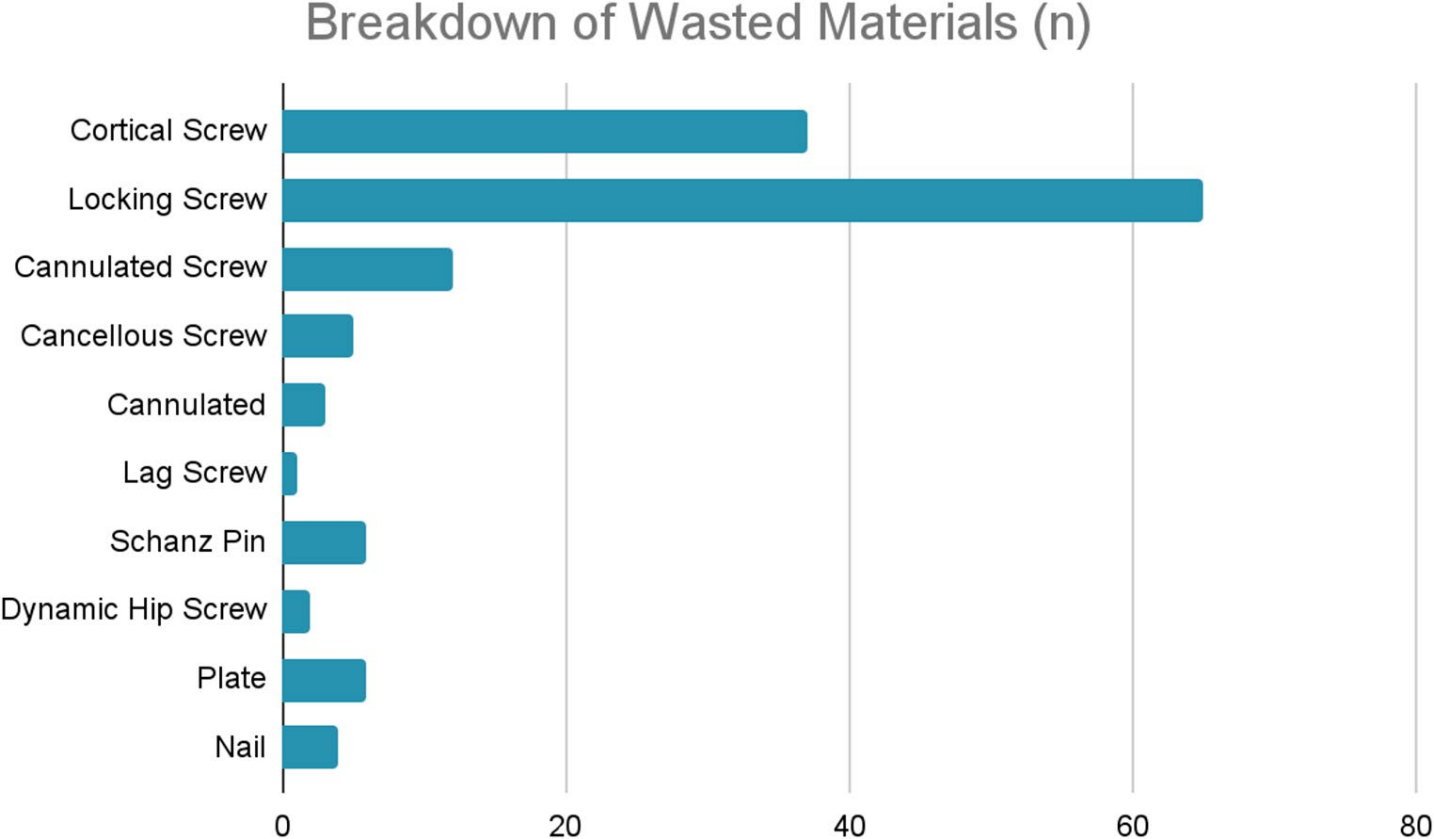
Distribution of implant wastage by material type

**Fig. 4:**
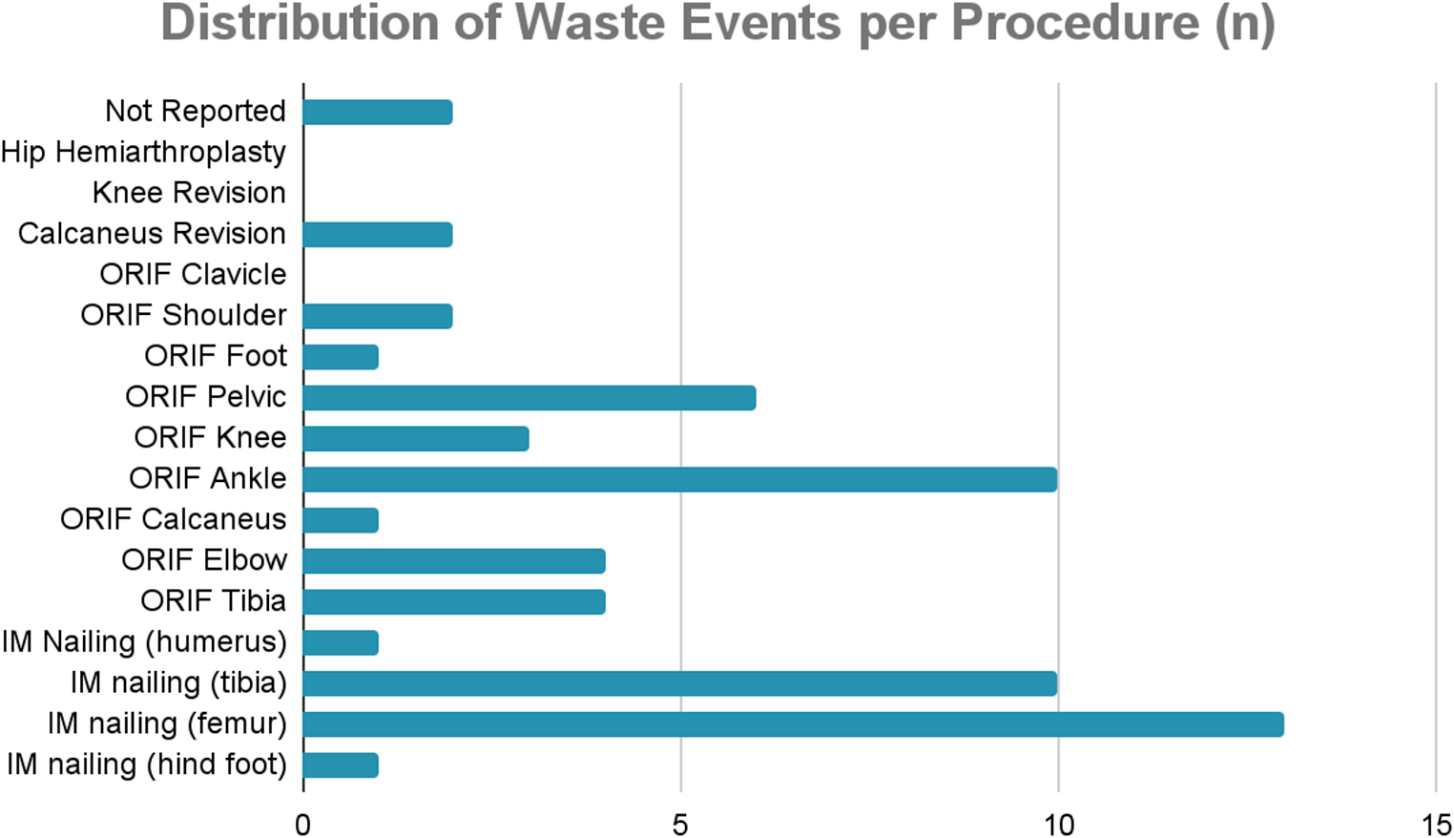
Frequency of implant wastage across different procedures

Across the 108 procedures with wastage, a total of 141 implants were wasted. Screws accounted for the vast majority of wastage, representing 131 (92.9%) of implants. Among these, locking screws were the most commonly discarded, with 65 screws wasted (46.1%), followed by cortical screws (37; 26.2%) and cannulated screws (12; 8.5%). Across ORIF and intramedullary nailing procedures, an overall screw wastage rate of 20% (17–31%) was observed with 2.4 screws wasted per trauma procedure. Other wasted implants included plates (6; 4.3%) and nails (4; 2.8%) (Figs. 3 and 4).

### Costs

Overall, the total cost of implant wastage during the study period was £14,744.

The total cost of wasted implants in orthopaedic trauma over the 44-day study period amounted to £335 per day and £136 per case. Locking screws, which accounted for the largest proportion of waste, also represented the highest cost, amounting to £7,475 - just over 50% of the total cost. Tables 1 and 2 provide a detailed breakdown of the implants wasted, their associated manufacturers and individual costs.

**Table 1:** Distribution of implant wastage across material types

Material	Quantity (*n*)	Percentage of Total Wastage (%)
Cortical Screw	37	26.2
Locking Screw	65	46.1
Cannulated Screw	12	8.5
Cancellous Screw	5	3.6
CCHS	3	2.1
Lag Screw	1	0.7
Schanz Pin	6	4.3
DHS	2	1.4
Plate	6	4.3
Nail	4	3.8

**Table 2:** Breakdown of the costs of implant wastage

Implant	Quantity (*n*)	Price per Item (£)	Cost (£)
Cortical Screw	37	35	1295
Locking Screw	65	115	7475
Cannulated Screw	12	129	1548
Cancellous Screw	5	48	240
Cannulated Compression Headless Screw	3	187	561
Lag Screw	1	37	37
Schanz Pin	6	42	252
Dynamic Hip Screw	2	84	168
Plate	6	261	1566
Nail	4	408	1632
Total			14,744

## Discussion

There are few studies assessing implant wastage in orthopaedic procedures at a UK hospital. Implant wastage rates were high and observed in 82.4% of procedures involving metalwork, with screws accounting for 92.9% of all wasted implants. Locking screws accounted for the highest wastage, comprising 46% of discarded screws and 51% of the total cost. Procedures such as intramedullary nailing of the femur, tibia, and ankle exhibited the highest wastage rates, likely reflecting the complexity of these surgeries.

The wastage rate of 82.4% exceeds the 33% reported in previous literature [12]. However, our findings align with previous reports indicating that screws are the most commonly wasted implant type [6]. This study found a rate of approximately 2.4 screws were wasted per trauma procedure that used metalwork. procedure. Jayakumar et al. attributed this trend to the frequent use of screws in trauma surgeries and the high likelihood of intraoperative over-selection. Similarly, Laurut et al. observed elevated wastage rates in procedures requiring multiple implants, such as hip arthroplasty, reinforcing the link between procedural complexity and wastage rates [13]. However, our study differs in identifying locking screws as the most frequently wasted type, whereas cortical screws are cited as the most commonly wasted in other studies [9].

Several factors likely contribute to our high wastage rates. Technical issues, including inaccurate depth gauge readings due to soft tissue interference, parallax errors in radiographic measurements, and limitations in imaging resolution, can lead to improper implant sizing and placement [14, 15]. Anatomical complexities, including intricate bone geometry and patient-specific variations, further complicate accurate measurements [16]. Notably, human and system factors, including the experience level of the surgical team and communication gaps, have been identified as significant contributors to implant wastage, with reports attributing up to 95% of such waste to these factors [6]. It is also worth mentioning that while the wastage rate was higher in nailing procedures (31%) compared to ORIFs (17%), the overall number of screws used in ORIFs was substantially greater, suggesting that the cumulative impact of wastage across both procedure types may be more balanced than the percentage difference alone implies.

The financial burden of implant wastage is substantial, with a total cost of £14,744 over the study period. This translates to an estimated annual cost of £86,561.55 in our centre and £2.4 million if similar wastage rates apply across the 27 major trauma centres in NHS England’s Major Trauma Network. Similarly, studies in Europe, such as the ones by Jayakumar et al. and Laurut et al., have also highlighted wastage rates as a key contributor to rising operative costs. Our figure of £14,744 is higher in value than the cost found by Jayakumar et al. at £8,377.25 [8]. Beyond its financial implications, implant wastage poses significant challenges to environmental sustainability. The production of medical implants, particularly screws and plates, is highly energy-intensive, contributing substantially to greenhouse gas emissions [17]. Stainless steel and titanium, the most commonly used materials for orthopaedic screw manufacturing, have notable carbon footprints [18]. Stainless steel production emits approximately 6.15 kg CO2e per kg, while titanium production is associated with even higher emissions and material wastage [19].

This study highlights the importance of targeted interventions to enhance precision surgery and economic sustainability in surgical practices. Key strategies include improving preoperative planning or developing precision-based technologies to minimise the over-selection of implants.

### Limitations

This study has several limitations that should be acknowledged. Firstly, as a retrospective study conducted at a single centre with a small sample size, the findings may not be generalisable to other NHS trusts or healthcare settings. Secondly, while the study effectively quantified implant wastage, it did not examine other contributors to surgical inefficiency. These factors likely influence overall implant usage and warrant further investigation in future studies. Future studies should employ a multi-centre design and integrate qualitative analyses to better identify the root causes of implant wastage, as well as variability across sites and regions.

## Conclusion

Implant wastage represents a significant financial and environmental burden in trauma and orthopaedic surgery. This study found screws, particularly locking screws, as the primary contributors to wastage. Implementing targeted interventions to reduce implant wastage could improve cost-efficiency and sustainability, facilitating resource optimisation and environmental responsibility.

## Data Availability

The datasets generated and analysed during the current study are available from the corresponding author upon reasonable request.
